# GBAP1 functions as a tumor promotor in hepatocellular carcinoma via the PI3K/AKT pathway

**DOI:** 10.1186/s12885-023-11107-7

**Published:** 2023-07-05

**Authors:** Rong Chen, Meng Zhao, Yanli An, Dongfang Liu, Qiusha Tang

**Affiliations:** 1 Department of Oncology, Zhongda Hospital, Medical School of Southeast University, Nanjing, 210009 Jiangsu Province China; 2 Medical college, Henan University of Traditional Chinese Medicine, 450001 Henan Province, China; 3 Jiangsu Provincial Laboratory of Molecular and Functional Imaging, Department of Radiology, Zhongda Hospital, Medical School of Southeast University, Nanjing, 210009 Jiangsu Province China; 4 Medical School of Southeast University, Nanjing, 210009 Jiangsu Province China

**Keywords:** GBAP1, Long non-coding RNA, Hepatocellular carcinoma, PI3K/AKT pathway

## Abstract

Hepatocellular carcinoma (HCC) is common worldwide, and novel therapeutic targets and biomarkers are needed to improve outcomes. In this study, bioinformatics analyses combined with in vitro and in vivo assays were used to identify the potential therapeutic targets. Differentially expressed genes (DEG) in HCC were identified by the intersection between The Cancer Genome Atlas and International Cancer Genome Consortium data. The DEGs were evaluated by a gene set enrichment analysis as well as Gene Ontology and Kyoto Encyclopedia of Genes and Genomes analyses. A protein interaction network, univariate Cox regression, and Lasso regression were used to screen out hub genes correlated with survival. Increased expression of the long noncoding RNA *GBAP1* in HCC was confirmed in additional datasets and its biological function was evaluated in HCC cell lines and nude mice. Among 121 DEGs, *GBAP1* and *PRC1* were identified as hub genes with significant prognostic value. Overexpression of *GBAP1* in HCC was confirmed in 21 paired clinical tissues and liver cancer or normal cell lines. The inhibition of GBAP1 expression reduced HCC cell proliferation and promoted apoptosis by inactivating the PI3K/AKT pathway in vitro and in vivo. Therefore, GBAP1 has a pro-oncogenic function in HCC and is a candidate prognostic biomarker and therapeutic target.

## Introduction

Hepatocellular carcinoma (HCC) is the fifth most common malignant tumor and the fourth most common cause of cancer-related deaths worldwide [1]. Surgery is the most effective treatment; however, only approximately 30–40% of patients can receive surgery at the time of diagnosis, and 44% of patients die within 2 years after surgery [2]. Other therapies include radiation therapy, transarterial chemoembolization, radiofrequency ablation, and various drugs and targeted therapies [3]. Some molecular targeted drugs have been approved for HCC treatment, such as the multikinase inhibitors sorafenib and lenvatinib, and the second-line drugs cabozantinib [4], regorafenib [5], and ramucirumab [6]. However, these drugs fail to substantially extend overall survival (OS) and progression-free survival, or improve quality-of-life [7]. Therefore, it is important to identify prognostic markers or therapeutic targets to improve clinical outcomes in HCC.

The diversity and dynamics of tumors make treatment difficult [8]. The establishment of highly specific biomarkers for the diagnosis, treatment, and prevention of diseases has been a major advance in cancer research [9, 10]. Identifying and validating predictive biomarkers can improve early diagnosis, treatment, and prognostic evaluation. The ultimate goal of molecular targeted therapy is to enhance the effectiveness and selectivity of cancer diagnosis and therapy by taking advantage of the differences between cancer tissues and normal tissues [11, 12].

The identification of differentially expressed genes (DEGs) and subsequent analyses of their biological effects is an effective approach for the development of diagnostic and prognostic biomarkers [13–15]. Long noncoding RNAs (lncRNAs) are a class of RNA molecules longer than 200 nucleotides that lack protein-coding function [16]. They function as either oncogenes or tumor suppressors in many types of cancers. In HCC, lncRNAs adjust the tumor microenvironment and affect various biological processes, including cell cycle progression, proliferation, invasion, apoptosis, and metastasis. Some studies have shown that lncRNAs influence HCC occurrence and progression via the notch, p53, VEGF, or Wnt signal transduction pathways. For example, the lncRNA ZEB1AS1 reduces HCC proliferation by targeting miR-365a-3p [17]. The knockdown of the lncRNA ASTILCS downregulates protein tyrosine kinase 2 (PTK2), leading to HCC cell death [18].

The *GBAP1* gene is an expressed GBA pseudogene located 16 kb downstream of the functional gene. Studies have shown [19] that GBAP1 may function as a competing endogenous RNA (ceRNA) to adjust GBA expression by sponging miR-22-3p in the pathogenesis of Parkinson’s disease or by binding competitively with miRNA-212-3p in gastric cancer [20]. It is a candidate biomarker for the diagnosis and prognosis of HCC; however, the effect and mechanism of action of the lncRNA GBAP1 in HCC are still unclear.

In this study, we evaluated DEGs in HCC and identified GBAP1 as a key lncRNA for further research. The expression and specific function of GBAP1 in HCC were then evaluated. Our results confirmed that the inhibition of GBAP1 suppresses proliferation and promotes apoptosis by inactivating the PI3K/AKT pathway.

## Materials and methods

### Differential gene expression analysis

Data of 374 liver hepatocellular carcinoma (LIHC) tissue samples and 50 normal liver tissues were downloaded from The Cancer Genome Atlas (TCGA, https://cancergenome.nih.gov/) [21]. The LICA-FR liver cancer data, including 150 liver cancer and 11 para-cancerous tissue samples from 150 patients with HCC, were acquired from the International Cancer Genome Consortium (ICGC; https://icgc.org) [22]. The GSE76427, GSE14520, GSE101685, GSE54236, and GSE64041 datasets were retrieved from Gene Expression Omnibus (GEO, https://www.ncbi.nlm.nih.gov/geo/) [23]. The GSE76427 dataset included data for 115 cancer tissue samples and 52 paracancerous samples from 115 patients with HCC based on GPL10558 (Illumina HumanHT-12 V4.0 Expression BeadChip) [24]. The GSE14520 dataset was developed from 222 cancer tissue samples and 212 paracancerous samples detected by the Affymetrix HT Human Genome U133A Array [25, 26]. The GSE101685 dataset contained expression profiling data obtained by Affymetrix Human Genome U133 Plus 2.0 from 8 normal tissues and 24 HCC cases. The GSE54236 dataset included data for 80 normal liver samples and 81 HCC samples [27] and GSE64041 included data for 60 biopsy pairs from patients with HCC plus five normal liver biopsies [28]. The differential expression analysis and visualization of results were performed using R (https://www.r-project.org/) with the limma and VennDiagram packages. The criteria for DEG identification were |log2 fold change (log2 FC)| > 1 and adjusted p < 0.05. Expression of GBAP1 in multiple tumors was evaluated using the Tumor IMmune Estimation Resource (TIMER, cistrome.shinyapps.io/timer) database.

### Functional enrichment analysis and PPI network

To annotate biological processes or pathways related to dysregulated genes in HCC, a Gene Ontology (GO) Biological Process enrichment analysis, Kyoto Encyclopedia of Genes and Genomes (KEGG) pathway enrichment analysis, and gene set enrichment analysis (GSEA) were performed using the clusterProfiler R package. For GSEA, “h.all.v7.1.symbols.gmt” from the Molecular Signatures Database (MsigDB; http://software.broadinstitute.org/gsea/msigdb/collections.jsp#H) was applied using the expression matrix of HCC samples from TCGA. A gene set was considered enriched when p < 0.05. Then, the intersecting DEGs were evaluated using the Search Tool for the Retrieval of Interacting Genes (STRING) (http://string-db.org; version: 11.0) in Cytoscape to build a protein–protein interaction (PPI) network and hub genes were identified using the plug-in cytoHubba.

### Survival analysis of hub genes

Hub genes strongly associated with prognosis and survival in HCC were screened out by a combination of LASSO Cox regression and univariate Cox regression analyses. Briefly, samples were classified into groups with low and high expression levels using the R package bestSeparation. A survival analysis was performed using the survival (https://github.com/therneau/surviva) and survminer packages (https://github.com/kassambara/survminer). Statistical significance was defined as log-rank p < 0.05. For GBAP1, univariate and multivariate Cox regression analyses were also performed.

### Cell culture

The immortalized human hepatocyte (MIHA) cell line was purchased from Lonza (Basel, Switzerland), the human HCC cell lines Huh7, Hep3B, and HepG2 were obtained from ATCC (Rockville, MD, USA), and MHCC-97 was obtained from the Chinese Academy of Sciences Cell Bank. Cells were cultivated in Dulbecco’s Modified Eagle Medium Thermo Fisher Scientific, Carlsbad, CA, USA) under standard conditions at 37 °C with 5% CO_2_. The 740Y-P reagent was purchased from MedChemExpress (Monmouth Junction, NJ, USA). Cells were incubated with 740Y-P at a final concentration of 10 µM for 24 h for subsequent experimental detection.

### Isolation of cytoplasmic/nuclear fractions

To separate the nuclear and cytoplasmic fractions, RNAs were detached using the Cytoplasmic and Nuclear RNA Purification Kit (Norgen Biotek Corp., Thorold, Canada) according to the manufacturer’s instructions. Approximately 1 × 10^7^ cells (HepG2) were lysed with lysis buffer on ice, and cytoplasmic or nuclear RNA was separated by centrifugation (14,000 × *g*) for 10 min, followed by q-PCR.

### Lentivirus construction and cell infection

The coding sequence of *GBAP1* was acquired from NCBI and used to design short hairpin RNAs (shRNAs) using RNAi Designer (Table 1). Next, shRNAs were cloned into the BamHI and EcoR I sites of the pLVX-puro vector. The lentiviral plasmid pLVX-shRNA2-puro and helper plasmids were co-transfected into 293T cells to generate lentiviral particles expressing the target fragment. The supernatant containing lentiviral particles was then harvested. Lentivirus infection was carried out using cells at 70% confluence with a multiplicity of infection (MOI) of 10 for HepG2 and Hep3B cells.

**Table 1:** shRNA sequences

shRNA	Sequence (5’-3’)
shGBAP1-1-forward	GATCCGCACCGGCACAGTGAAATAAGCTCGAGCTTATTTCACTGTGCCGGTGCTTTTTG
shGBAP1-1-reverse	AATTCAAAAAGCACCGGCACAGTGAAATAAGCTCGAGCTTATTTCACTGTGCCGGTGCG
shGBAP1-2-forward	GATCCGCACAGTGAAATAAGATTTCGCTCGAGCGAAATCTTATTTCACTGTGCTTTTTG
shGBAP1-2-reverse	AATTCAAAAAGCACAGTGAAATAAGATTTCGCTCGAGCGAAATCTTATTTCACTGTGCG

### Cell counting Kit-8 (CCK-8) assay

The CCK-8 assay (96992-100TESTS-F, Sigma, St. Louis, MO, USA) was performed to assess cell growth. Approximately 5 × 10^3^ cells per well were added to 96-well microplates. Then, 10 µl of CCK-8 was added to the cells for 1 h. Absorbance was evaluated at 450 nm for each well using a microplate reader (PerkinElmer, VICTOR NIVO, Waltham, MA, USA). All experiments were performed in triplicate.

### EdU cell proliferation assay

Cells in the logarithmic growth phase were seeded in 96-well plates (4 × 10^3^~1 × 10^5^ cells/well) and 100 µL of 50 µM EdU medium was added to each well. Cells were fixed, washed with PBS, and 100 µL of 1×Apollo staining reaction solution was added to each well. The cells were then washed with PBS and 100 µL of 1×Hoechst33342 reaction solution was added to each well for DNA staining. The cells were observed under a fluorescence microscope and the EdU positive rate (number of EdU positive cells/total cells) was determined.

### Apoptosis assays

The Annexin V-FITC/PI Double Staining Kit (Jiangsu KeyGEN, KGA108-1, Nanjing, China) was used to detect apoptotic cells by flow cytometry, as described previously [29]. Briefly, positively stained cells (apoptotic cells) were quantified (ACEA Bio, 2040R; San Diego, CA, USA) and analyzed using FlowJo-V10.

### q-PCR analysis

Total RNA from tissue or cells was extracted with TRIzol reagent (Thermo Fisher Scientific, 15,596,026) following the manufacturer’s instructions. Complementary DNA (cDNA) was synthesized and PCR with cDNA templates was performed using a real-time detector (Analytik Jena AG, qTower 3.2G; Jena, Germany) using BeyoFast SYBR Green qPCR Mix (Bio-Rad, 1708882AP, Shanghai, China). The primer sequences were shown in Table 2. Transcript levels were normalized against *GAPDH* levels as an internal reference and were evaluated using the 2^−ΔΔCt^ method. All experiments were repeated three times.

**Table 2:** Probe sequences

Primer	Sequence (5’to3’)^*^
GAPDH Forward	5′-ACAGCCTCAAGATCATCAGC-3′
GAPDH Reverse GBAP1 Forward GBAP1 Reverse	5′-GGTCATGAGTCCTTCCACGAT-3′ 5′-TGCTTCTACTTCAGGCAGTGTCG-3′ 5′-CTTTCTGAGCCTGAGTCCGTAGC-3′

* Both sides are labeled with biotin

**Table 3:** Univariate and multivariate Cox regressions on clinicopathological characteristics and GBAP1

Variables	Univariate Cox			Multivariate Cox	
	HR (95% CI)	p value		HR (95% CI)	p value
**gender**	0.816(0.573–1.163)	0.260		1.001(0.671–1.493)	0.997
**age**	1.010(0.997–1.024)	0.139		1.013(0.998–1.029)	0.097
**Stage**	2.676(1.754–4.083)	<0.001		2.267(1.458–3.526)	<0.001
**metastasis**	2.479(1.695–3.897)	0.005		4.347(2.738–6.535)	0.002
**GBAP1**	1.429(1.225–1.668)	<0.001		1.389(1.166–1.655)	<0.001

### Western blot assay

The cells or tissues were lysed in 2× sodium dodecyl sulfate (SDS) lysis buffer. Protein concentrations were measured using the BCA Protein Concentration Assay Kit (Beyotime, Shanghai, China) and sodium dodecyl sulfate polyacrylamide gel electrophoresis (SDS-PAGE) was used to separate the cell lysates. The samples were moved to a polyvinylidene difluoride (PVDF) membrane (Millipore, Billerica, MA, USA). The membranes were incubated with primary antibodies against PCNA, MCM2, PI3K, p-PI3K, AKT, and p-AKT (1:1000; ABM, Vancouver, Canada) and GAPDH as a reference (1:6,000; Proteintech, Wuhan, China) overnight at 4 °C. The membrane was then incubated with a horseradish peroxidase (HRP)-conjugated anti-mouse or anti-rabbit secondary antibody (CST, Danvers, MA, USA) for 2 h at 25 °C. Signals were detected using the Enhanced Chemiluminescence Kit (NCM Biotech, Suzhou, China) and a chemiluminescence imaging system (Tanon, Shanghai, China). As there were many target proteins, the different incubation conditions of each antibody lead to different gel cutting and exposure time. In order to develop and expose in time, we cut it independently. As a result, different films were not exposed together.

### Xenograft mouse model

Female BALB/c nude mice (5 to 6 weeks old) weighing approximately 18–20 g were acquired from the Shanghai Experimental Animal Center, Chinese Academy of Sciences (Shanghai, China). Mice were maintained in a specific pathogen-free (SPF) animal facility. Twelve mice were randomly classified into two groups: sh-lncRNA-GBAP1 and NC (n = 6 each). Approximately 5 × 10^6^ cells were subcutaneously injected into the oxter of each mouse. The subcutaneous tumor length and width were determined every 4 days. The tumor size was calculated as follows: bulk (m^3^) = 1/2 length × width^2^. All mice were euthanized 30 days after inoculation.

### Immunohistochemical staining

Surgically resected tumor samples were fixed in formaldehyde and embedded in paraffin. The samples were washed with PBS three times, and 5% bovine serum albumin was used to block non-specific reactions. Antibodies against MCM2 (1:200 dilution) and PCNA (1:300 dilution) were added, followed by incubation at 4 °C overnight. Then, the slides were stained with 3,3′-diaminobenzidine (DAB) and counterstained with hematoxylin according to the manufacturer’s protocol. Images were acquired using a Nikon Eclipse 80i microscope (Nikon Americas Inc., Melville, NY, USA).

### Clinical samples

In total, 21 pairs of fresh liver cancer tissues and adjacent normal tissues were acquired between January 2021 and April 2021 from patients with HCC who did not receive any preoperative anti-tumor treatment or surgery at Jiangsu Provincial People’s Hospital. All experiments were approved by the Ethics Committee (2021ZDSYLL245-P01).

### Statistical analysis

Statistical analyses were performed using GraphPad Prism 8.0. The log-rank test was applied to assess differences between survival curves. For cell and animal experiments, Student’s *t*-tests were used for comparisons between two groups. Univariate comparisons among multiple groups were performed by one-way analysis of variance (ANOVA) followed by Dunnett’s tests. Statistical significance was set at *P* < 0.05.

## Results

### Screening of differentially expressed genes

**Fig. 1:**
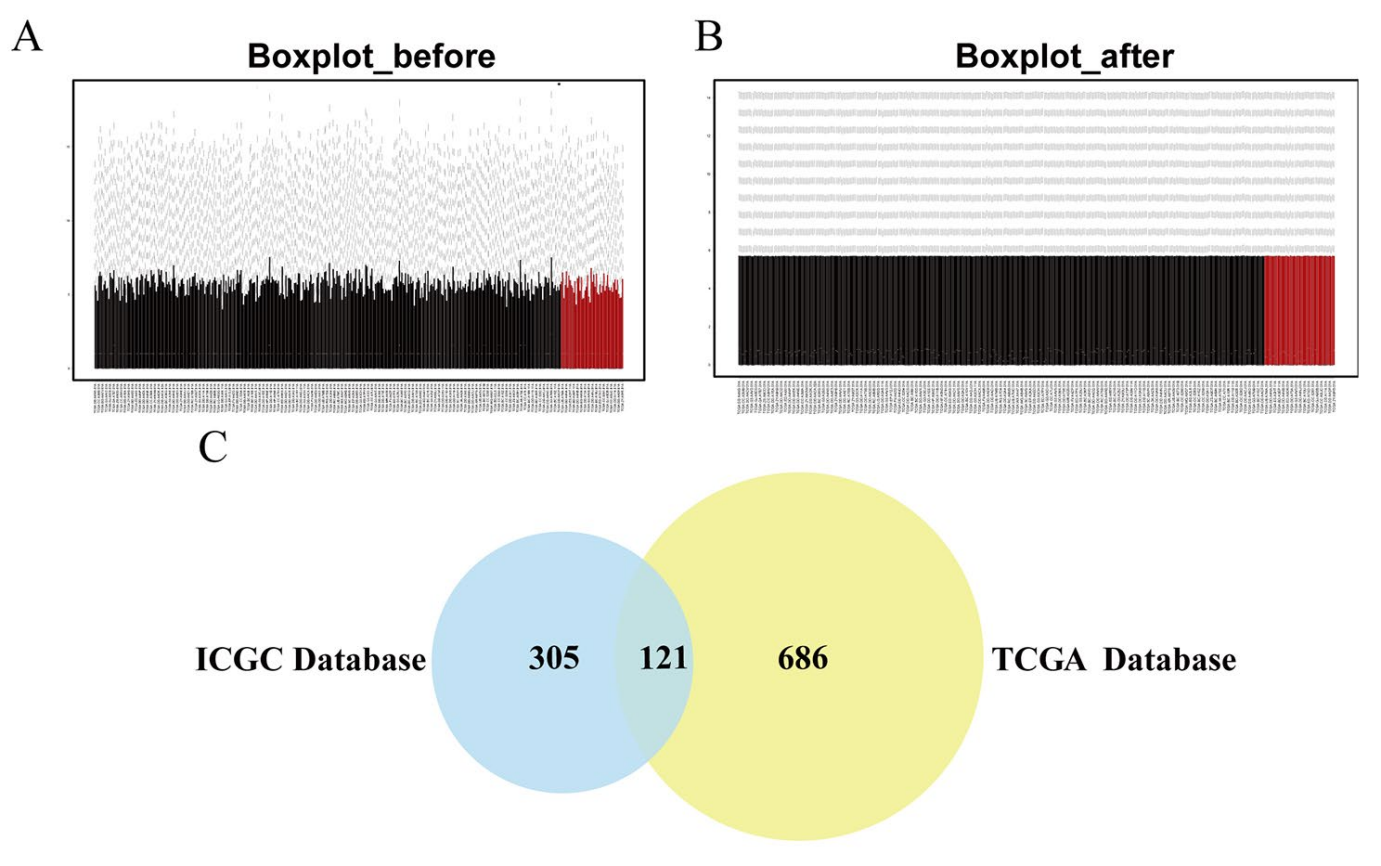
Identification of differentially expressed genes (DEGs) in liver cancer (LIHC) **A** Boxplot of Intergroup Distribution before Matrix Correction **B** Boxplot of Intergroup Distribution after Matrix Correction **C** Venn diagram demonstrates the intersections of DEGs between The Cancer Genome Atlas (TCGA) and International Cancer Genome Consortium (ICGC) data In figure A and B, the ordinate represents the mean value of gene expression, and the abscissa represents a patient sample

Using the limma package to preprocess the original expression data in the TCGA dataset, the mean gene expression levels in each sample were found to be essentially the same. These results show that data homogenization was successful, and the sample data source was reliable (Fig. 1A, B). We identified 2207 DEGs in TGCA and 926 DEGs in the ICGC database, and the intersection included 121 DEGs, as shown in a Venn diagram in Fig. 1C. The original data analyzed by TCGA and ICGC were analyzed using limma packets after data normalization processing. The interception criteria for DEGs in the above databases were all |log2 fold change(log2FC)|>1 and adjust P < 0.05.

### Functional enrichment analyses

**Fig. 2:**
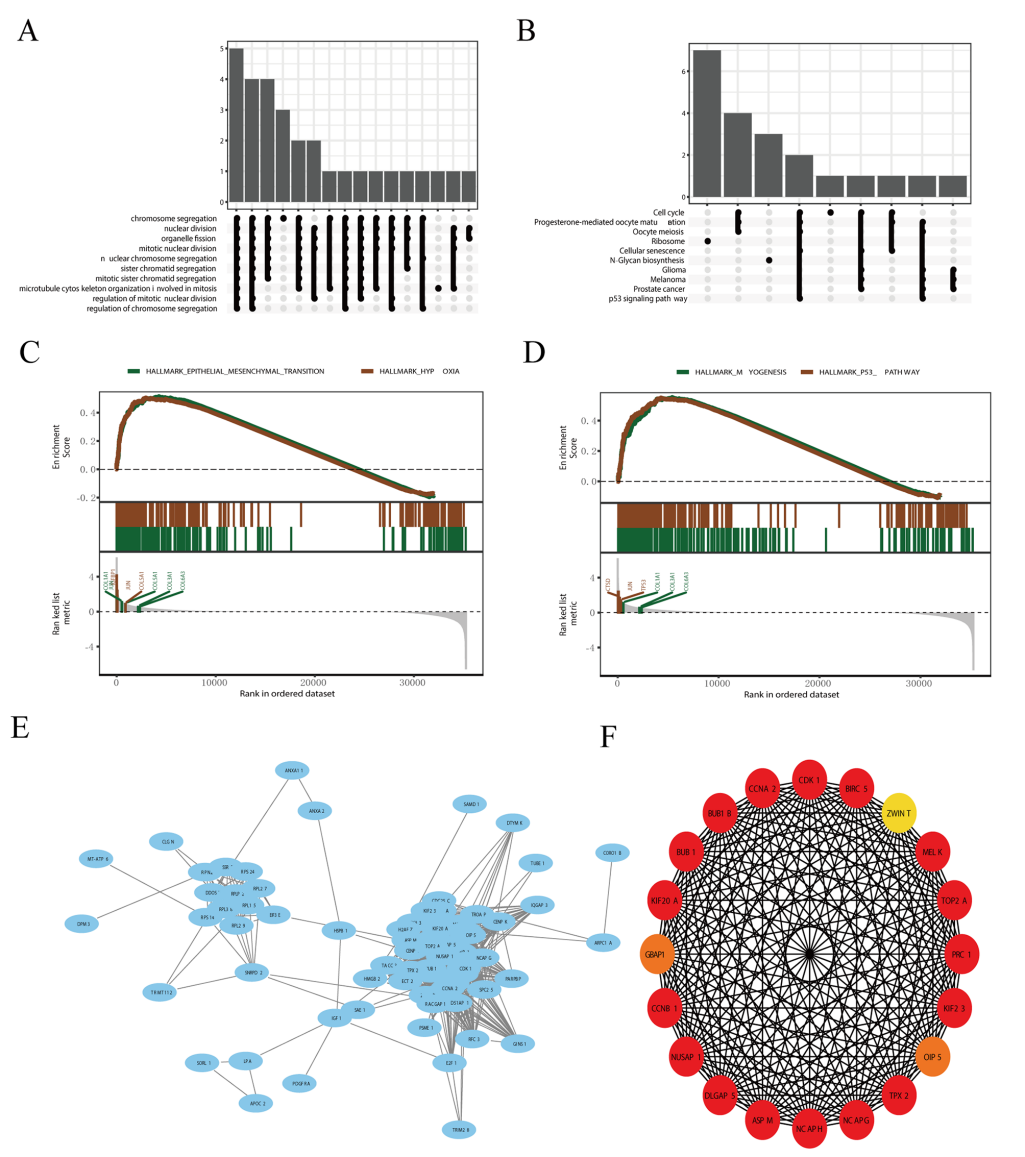
Analysis of differentially expressed genes (DEGs) **A** Gene ontology (GO) analysis of DEGs. **B** Kyoto Encyclopedia of Genes and Genomes (KEGG) analysis of DEGs. **C**, **D** Gene set enrichment analysis (GSEA) of liver hepatocellular carcinoma (LIHC)-related genes **E** The protein–protein interaction (PPI) network of DEGs was generated using network analyst **F** The top 20 hub genes associated with LIHC

We conducted a functional enrichment analysis of DEGs. A GO analysis revealed that DEGs are mainly enriched in mitotic nuclear division, nuclear division, mitotic sister chromatid segregation, chromosome segregation, nuclear chromosome segregation, and organelle fission (Fig. 2A). A KEGG pathway analysis showed that DEGs are mainly enriched in oocyte meiosis, progesterone-mediated oocyte maturation, ribosomes, and cell cycle pathways (Fig. 2B). The GSEA does not require significant differences in gene expression and can retain genes with little expression changes but important functions, thereby providing more information than traditional GO and KEGG enrichment analyses. The GSEA showed that the DEGs, particularly *COL1A1, COL3A1, COL5A1, COL6A3, JUN*, and *IGFBP1*, were mainly involved in the epithelial mesenchymal transition signaling pathway and hypoxia (Fig. 2C). In addition, *CTSD, JUN, TP53, COL1A1, COL3A1*, and *COL6A3*, were involved in MYOGENESIS and P53_PATHWAY signaling pathways (Fig. 2D).

Based on the 121 DEGs, we constructed a PPI network and performed a module analysis (Fig. 2E). As shown in Figs. 2F and 20 candidate hub genes were obtained.

### Hub gene survival analysis

**Fig. 3:**
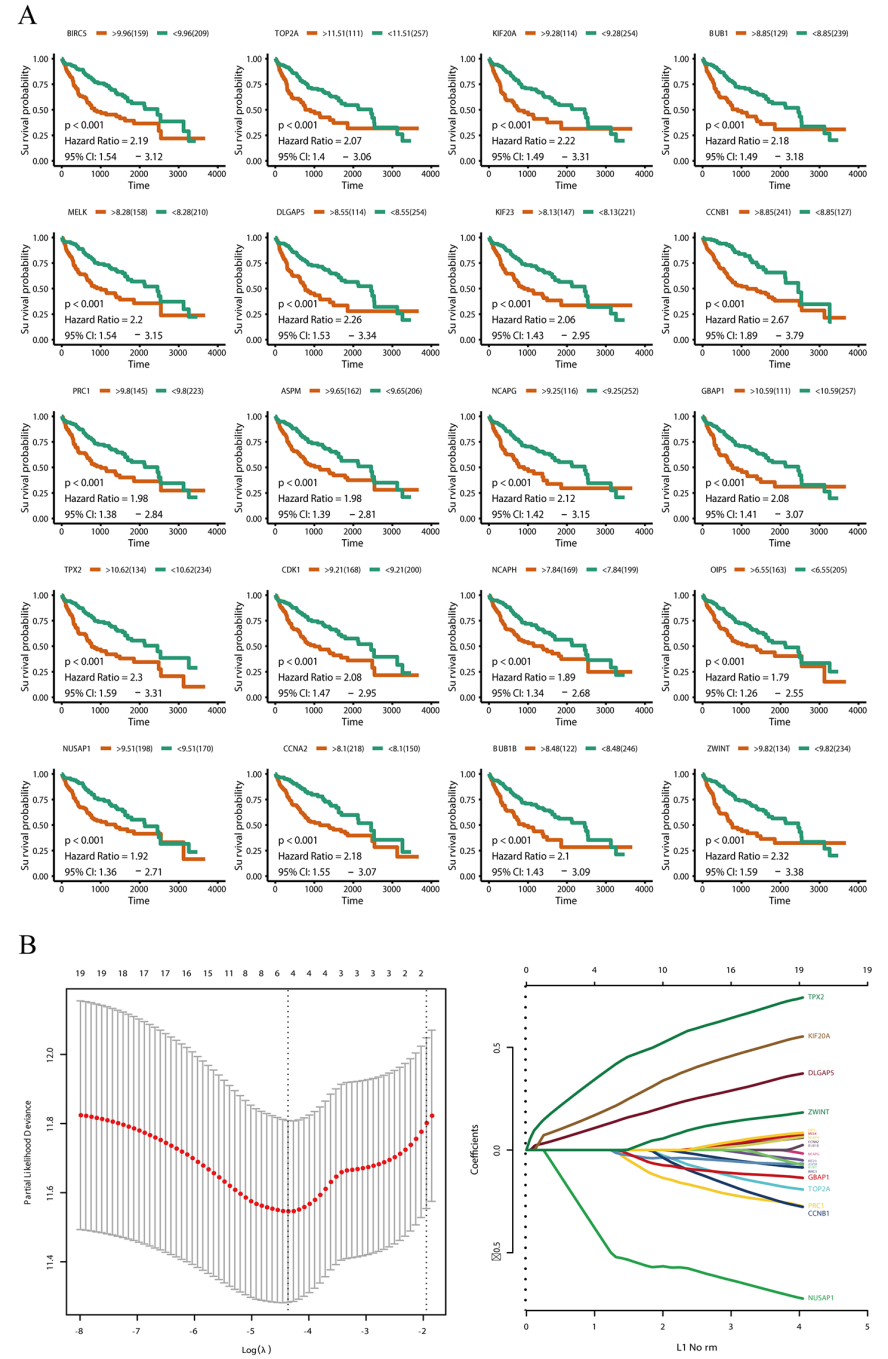
Hub genes survival analysis **A** Kaplan–Meier survival curves for hub genes in The Cancer Genome Atlas (TCGA) cohorts. The horizontal axis indicates the overall survival time in days, and the vertical axis indicates the survival rate **B** LASSO Cox survival analysis

To investigate the relationships between hub genes and survival in LIHC, we performed univariate Cox regression and LASSO Cox regression analysis. Based on Kaplan–Meier analyses, the 20 hub genes were all related to prognosis in LIHC (Fig. 3A). The LASSO Cox regression screening variables are shown in Fig. 3B. These results indicate that *GBAP1* and *PRC1* may contribute to abnormal signaling in LIHC and are candidate prognostic biomarkers. The *GBAP1* gene is an lncRNA; accordingly, we focused on the role of *GBAP1* in the development and progression of HCC.

### GBAP1 expression and functional enrichment

**Fig. 4:**
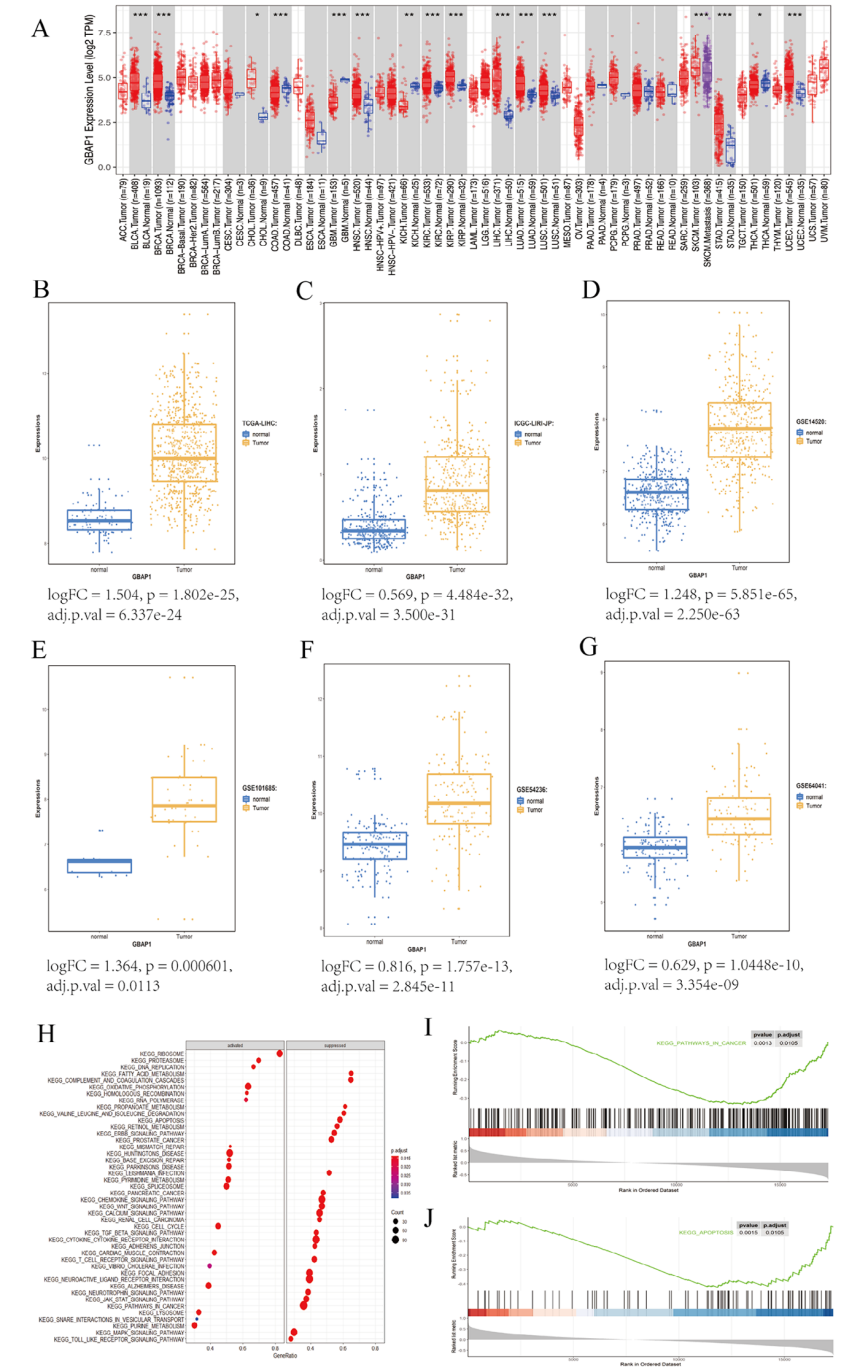
GBAP1 expression and functional enrichment **A** Differential expression of prognostic markers GBAP1 in various cancers **B** Differential expression of lncRNA GBAP1 in The Cancer Genome Atlas liver hepatocellular carcinoma (TCGA-LIHC) data: logFC = 1.504, p = 1.802e-25, adj.p.val = 6.337e-24 **C** Differential expression of long noncoding RNA (lncRNA) GBAP1 in ICGC-LIRI-JP data: logFC = 0.569, p = 4.484e-32, adj.p.val = 3.500e-31 **D** Differential expression of core difference lncRNA GBAP1 in GSE14520 data: logFC = 1.248, p = 5.851e-65, adj.p.val = 2.250e-63 **E** Differential expression of core difference lncRNA GBAP1 in GSE101685 data: logFC = 1.364, p = 0.000601, adj.p.val = 0.0113 **F** Differential expression of core difference lncRNA GBAP1 in GSE54236 data: logFC = 0.816, p = 1.757e-13, adj.p.val = 2.845e-11 **G** Differential expression of core difference lncRNA GBAP1 in GSE64041data: logFC = 0.629, p = 1.0448e-10, adj.p.val = 3.354e-09 **H** Kyoto Encyclopedia of Genes and Genomes gene set enrichment analysis (KEGG- GSEA) **I** KEGG analysis of pathways in Cancer **J** KEGG analysis of Apoptosis (p.adjust < 0.05)

In a pan-cancer analysis, compared with levels in normal control groups, *GBAP1* expression was increased in liver cancer, breast cancer, kidney cancer, bowel cancer, and others (Fig. 4A). Expression of *GBAP1* was significantly up-regulated in cancer tissues in additional datasets: The Cancer Genome Atlas liver hepatocellular carcinoma (TCGA-LIHC) (Fig. 4B), ICGC-LIRI-JP, which was the RNAseq data of liver cancer expression sample provided by RIKEN (JP) in Japan (Fig. 4C), GSE14520 (Fig. 4D), GSE101685 (Fig. 4E), GSE54236 (Fig. 4F), and GSE64041 (Fig. 4G). A univariate Cox regression analysis using the TCGA cohort confirmed that high GBAP1 expression is associated with a worse prognosis based on OS (Table 3). In a multivariate Cox regression analysis, high GBAP1 expression was also related to a worse OS, suggesting that GBAP1 expression is an independent prognostic factor for OS (*P* < 0.001; Table 3).

Next, KEGG-GSEA was performed to analyze changes in biological processes related to GBAP1. The top 20 significantly activated or suppressed gene sets are shown in Fig. 4H. Notably, we also observed the enrichment of pathways related to cancer and apoptosis (Fig. 4I–J).

### GBAP1 is highly expressed in HCC and its inhibition reduces cell proliferation

**Fig. 5:**
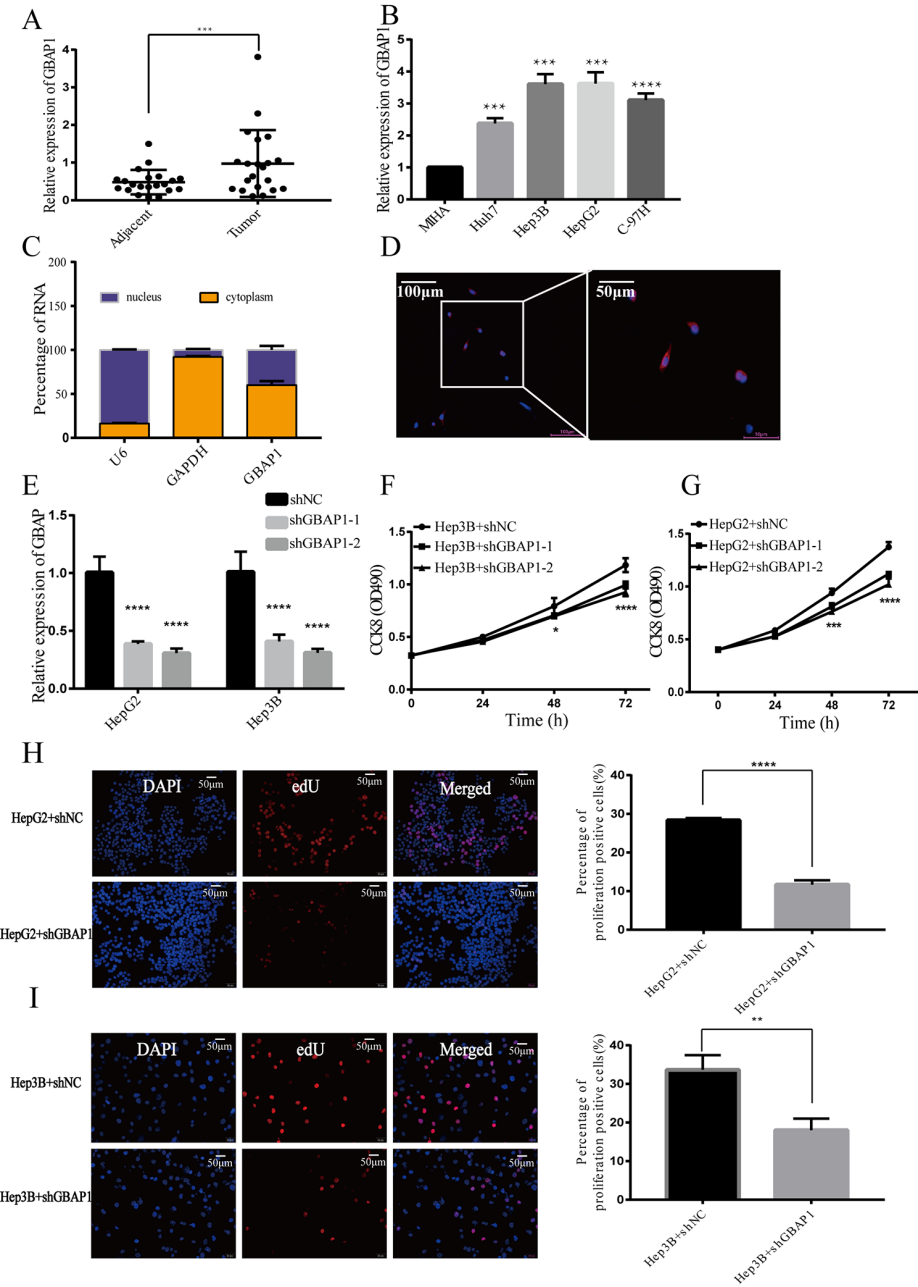
GBAP1 is highly expressed in liver cancer cells and inhibiting its expression reduces cell proliferation **A** Differential expression of GBAP1 in 21 cancer tissue pairs and adjacent tissues of patients with liver cancer **B** Expression of GBAP1 in hepatocellular carcinoma (HCC) cell lines **C** The percentage of GBAP1 in nucleus and cytoplasm **D** Subcellular localization of GBAP1 **E** GBAP1 was significantly silenced in HepG2 cells and Hep3B cells **F** Cell Counting Kit-8 (CCK8) analysis of Hep3B cell growth **G** CCK8 analysis of HepG2 cell growth **H** EdU assay of HepG2 **I** EdU assay of Hep3B

Analyses of 21 paired cancer and adjacent tissues from patients with HCC indicated that the expression of GBAP1 in HCC tissues is remarkably higher than that in adjacent tissues (*P* < 0.001) (Fig. 5A). To assess the expression pattern of GBAP1 in HCC cell lines (HepG2, Hep3B, Huh7, and MHCC-97 H) and normal liver cells (MIHA), q-PCR was conducted. As shown in Fig. 5B, GBAP1 was highly expressed in the Huh7, HepG2, Hep3B and MHCC-97 H cell lines. We further evaluated its subcellular localization and found that in HepG2 cells, GBAP1 was highly enriched in the cytoplasmic fraction (Fig. 5C, D). To further study the function of GBAP1 in HCC, cell lines with a stable knock-down of GBAP1 were constructed by the transduction of HepG2 and Hep3B, using a lentivirus constitutively expressing either shNC or shGBAP1. Expression of GBAP1 was significantly silenced in HepG2 cells and Hep3B cells (Fig. 5E). We evaluated the impact of GBAP1 on the growth of HepG2 cells and Hep3B cells in vitro. The CCK-8 assays revealed that GBAP1 knockdown inhibited Hep3B (Fig. 5F) and HepG2 (Fig. 5G) cell growth compared with that in the control group. The EdU assay showed that the proliferation ability of HepG2 and Hep3B cells were also reduced after GBAP1 knockdown (Fig. 5H, I).

**Fig. 6:**
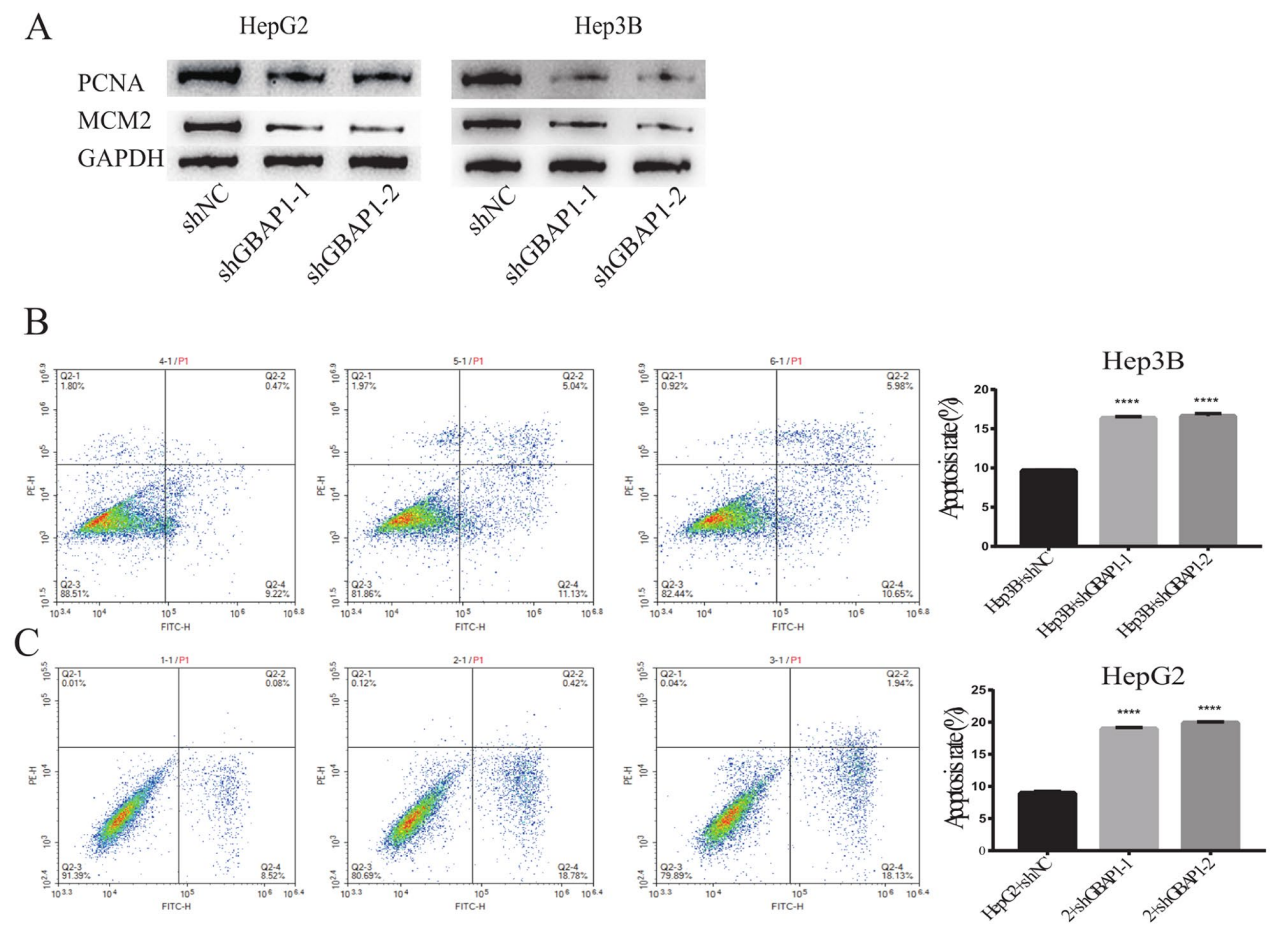
Inhibition of GBAP1 expression reduces cell proliferation markers and promotes cell apoptosis **A** Western blot analysis of MCM2 and PCNA **B** Apoptosis rates of GBAP1-knockdown Hep3B cells **C** Apoptosis rates of GBAP1-knockdown HepG2 cells

Levels of proliferation markers MCM2 and PCNA were lower in cells with stable GBAP1 knockdown than that in control cells (Fig. 6A). Annexin V/PI staining was applied to assess the effect of GBAP1 on apoptosis. The inhibition of GBAP1 significantly increased apoptosis (Fig. 6B, C). These results indicate that GBAP1 may have an oncogenic function in HCC.

### GBAP1 regulates HCC growth via the PI3K/AKT signaling pathway

**Fig. 7:**
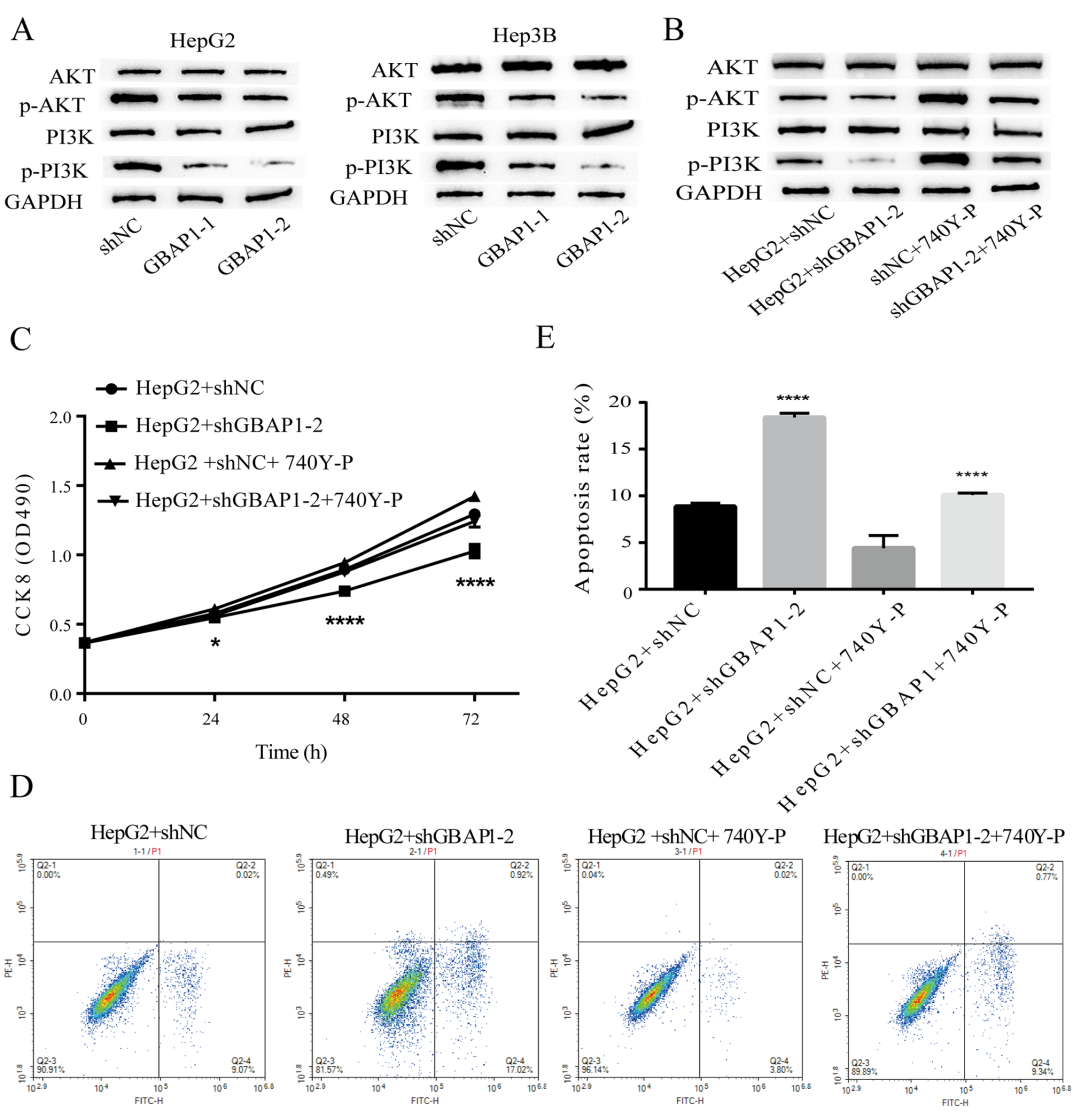
Inhibition of GBAP1 inhibits cell proliferation, and increases apoptosis through blocking the PI3K/AKT signaling pathway **A** GBAP1 knockdown decreased the phosphorylation levels of AKT and PI3K **B** Decreasing phosphorylation levels of AKT and PI3K by GBAP1 knockdown was rescued by the PI3K/AKT pathway activator 740Y-P. **C** Inhibition of growth by GBAP1 knockdown was reversed by the PI3K/AKT pathway activator 740Y-P. **D**, E Inducing apoptosis by GBAP1 knockdown was reversed by the PI3K/AKT pathway activator 740Y-P.

The dysregulation of PI3K/AKT signaling is a common feature of tumors [30, 31] and plays a major role in regulating cell growth and apoptosis [32–34]. We evaluated the protein levels of PI3K/AKT and the phosphorylated form (p-AKT/PI3K) after GBAP1 knockdown by western blotting. The knockdown of GBAP1 decreased the phosphorylation of AKT and PI3K (Fig. 7A). To confirm whether GBAP1 regulates HCC growth via the PI3K/AKT signaling pathway, a rescue experiment was performed. The PI3K/AKT pathway activator 740Y-P effectively increased PI3K/AKT signaling (marked by phosphorylated PI3K/AKT) (Fig. 7B) and promoted growth (Fig. 7C). One-way analysis of variance was conducted to compare CCK8 of the four groups at 72 h, and the results showed that compared with HepG2 + shGBAP1-2, CCK8 in HepG2 + ShgBAP1-2 was significantly decreased (P < 0.0001), while CCK8 in HepG2 + shNC + 740Y-P was significantly increased (P < 0.05). CCK8 in HepG2 + shGBAP1-2 + 740Y-P was significantly increased compared to HepG2 + shGBAP1-2 (P = 0.0005). 740Y-P also inhibited apoptosis (Fig. 7D, E) in 740Y-P-treated liver cancer cells with GBAP1 knockdown. These results demonstrated that GBAP1 accelerated liver cancer progression via the regulation of the PI3K/AKT signaling pathway.

### GBAP1 suppression inhibits HCC growth in vivo

**Fig. 8:**
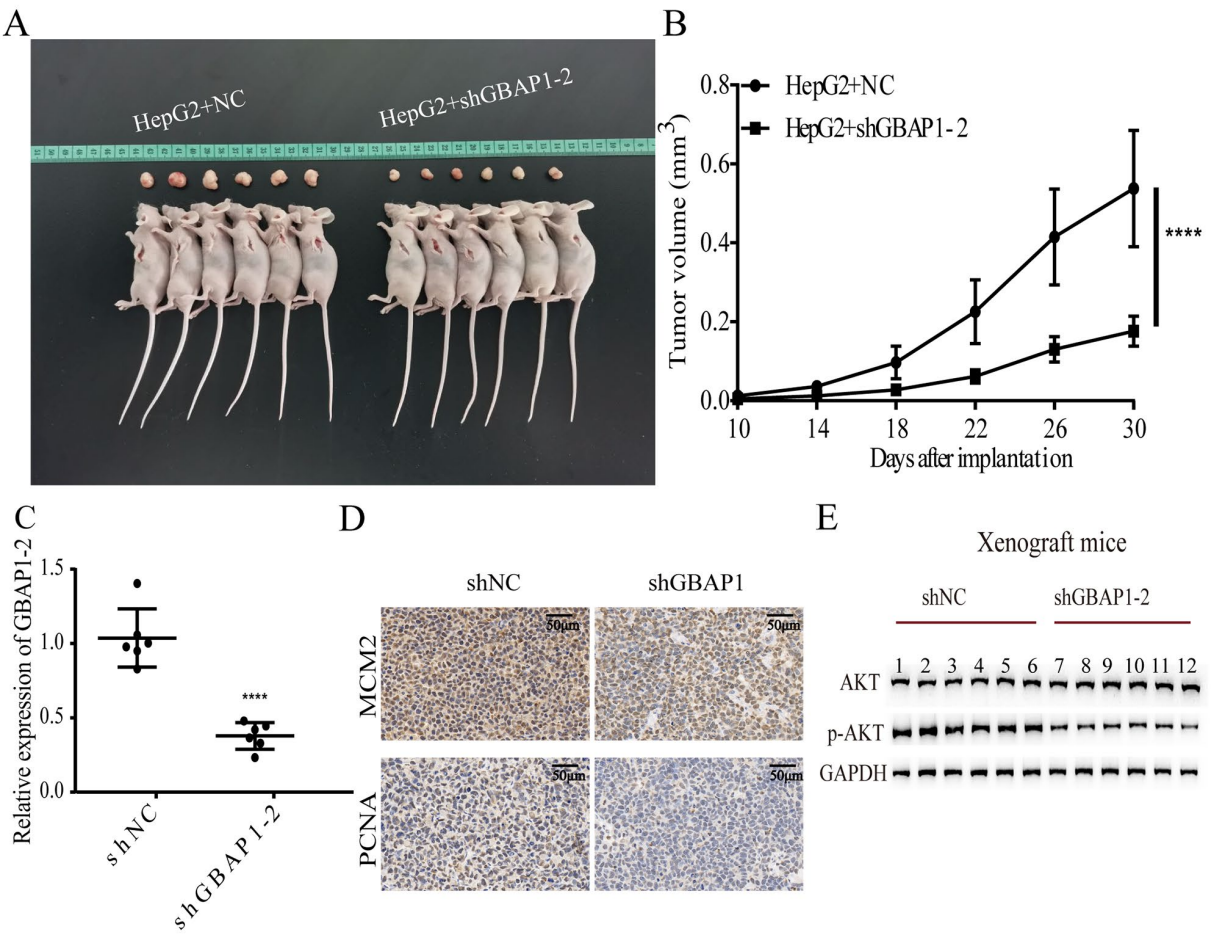
Inhibition of GBAP1 in vivo decelerates tumor growth through the PI3K/AKT pathway **A** GBAP1 knockdown inhibited tumor growth in vivo **B** Tumor volume was reduced in both shGBAP1 groups **C** Silencing of GBAP1 was confirmed in LV-shGBAP1-2 group **D** IHC(immunohistochemistry) of MCM2 and PCNA staining in HCC tissue **E** Western blot analysis of AKT and p-AKT

To further assess the effects of GBAP1 on tumorigenicity, a mouse model was utilized. As shown in Fig. 8A, tumor growth was significantly lower in mice with GBAP1 knockdown than that in the NC group. The tumor volume was lower in shGBAP1 groups than that in the NC group (Fig. 8B). Additionally, a q-PCR analysis confirmed that *GBAP1* expression levels were lower in the shGBAP1-2 group than that in the NC group (Fig. 8C). Expression levels of *MCM2* and *PCNA* in xenograft tumor tissues were lower in the shGBAP1-2 group than that in the NC group (Fig. 8D). A western blot analysis indicated that the p-AKT/AKT ratio was reduced in the shGBAP1 group (Fig. 8E). These results were consistent with those of in vitro assays and demonstrated that the GBAP1 knockdown inhibits HCC growth by inactivating the PI3K/AKT signaling pathway.

## Discussion

The 5-year recurrence rate is approximately 80% for patients with HCC who undergo resection [35], and new strategies for early diagnosis and treatment are urgently needed [36]. Owing to the lack of symptoms at the early stage, most patients with HCC have advanced disease at the time of diagnosis, with limited therapies [37]. Moreover, drug resistance, recurrence, and metastasis contribute to a poor prognosis [38, 39]. Therefore, studies of the mechanism underlying HCC are needed.

Genetic and epigenetic changes altering downstream signaling pathways lead to hepatocarcinogenesis [40–42]. Alterations in lncRNA expression are associated with tumor occurrence, development, chemotherapy resistance, metastasis, and recurrence; therefore, lncRNAs are potential diagnostic and therapeutic targets [43, 44]. The oncogenes *RAS* and *MYC* participate in the development and progression of tumors via the lncRNAs Orilnc1 and DANCR [45, 46]. In pancreatic cancer, levels of the lncRNA HULC are related to tumor volume, vascular infiltration, and OS [47] .

The GBAP1 acts as a ceRNA to adjust GBA expression by sponging miR-22-3p in the pathogenesis of Parkinson’s disease [48]. High GBAP1 expression is associated with a poor prognosis in HCC [49]. A cluster of lncRNAs (C3P1, GBAP1, HNF4A-AS1, and DIO3OS) function as ceRNAs in the occurrence and progression of HCC, and may be effective biomarkers for diagnosis and the prediction of prognosis and metastasis in patients with HCC [50]. However, the mechanism underlying the effects of GBAP1 in HCC have not been established.

In this study, we found 121 differentially expressed genes in HCC, including the long non-coding RNA pseudogene *GBAP1*. Analyses of four GEO datasets, multi-set chip data, sequencing data, and 21 paired cancer tissues and adjacent tissues in patients with HCC all confirmed that GBAP1 expression is increased in liver cancer tissues.

We verified, in vitro, that the lncRNA GBAP1 tends to be increased in HCC cell lines. The knockdown of GBAP1 inhibited HepG2 and Hep3B cell growth, and decreased the expression of the proliferation markers MCM2 and PCNA. Moreover, GBAP1 knockdown increased the percentage of apoptotic cells. We further confirmed, in vivo, that stable GBAP1 knockdown inhibits tumor growth in mice and reduces tumor weight. These results suggest that GBAP1 promotes HCC development.

The PI3Ks are heterodimeric lipid kinases consisting of a regulatory and catalytic subunit, encoded by diverse genes [51, 52]. The AKT protein is a downstream PI3K effector able to regulate a number of biological processes, including apoptosis and proliferation. Many studies have shown that the activation of the PI3K/AKT signaling pathway plays a vital role in regulating the progression and metastasis of many types of cancer, including breast cancer and HCC [3, 32, 53]. The inactivation of this pathway results in reduced cell proliferation and increased apoptosis [33, 54–56]. Changes in SVEP1, mediated by MiR-1269b, cause HCC proliferation and metastasis, probably via the PI3K/AKT pathway [57]. Gong et al. suggested that the tumorigenic function of NCAPG in HCC depends on the PI3K/AKT signaling pathway [34]. Liao et al. indicated that apatinib increases the radiosensitivity of HCC cells by inhibiting PI3K/AKT signaling [58].

We found that GBAP1 knockdown inactivates the PI3K/AKT pathway. The PI3K/AKT pathway activator 740Y-P effectively increased PI3K/AKT signaling, increased growth, and inhibited apoptosis in HCC cells with GBAP1 knockdown.

In brief, we found that the lncRNA GBAP1 is commonly overexpressed in HCC. Hence, inhibition of GBAP1 suppresses proliferation and promotes apoptosis by inactivating the PI3K/AKT pathway. Based on these results, GBAP1 is an effective candidate biomarker for tumor progression and relapse and for the clinicopathological diagnosis of HCC.

## Data Availability

The datasets used in the current study are available in the https://cancergenome.nih.gov/.
